# Homologous Recombination in *Clostridioides difficile* Mediates Diversification of Cell Surface Features and Transport Systems

**DOI:** 10.1128/mSphere.00799-20

**Published:** 2020-11-18

**Authors:** Hannah D. Steinberg, Evan S. Snitkin

**Affiliations:** a University of Michigan School of Public Health, Ann Arbor, Michigan, USA; b Department of Microbiology and Immunology, University of Michigan, Ann Arbor, Michigan, USA; Baylor College of Medicine

**Keywords:** *Clostridioides difficile*, S layer, flagella, homologous recombination, phosphotransferase system

## Abstract

Illness caused by the pathogen Clostridioides difficile is widespread and can range in severity from mild diarrhea to sepsis and death. Strains of C. difficile isolated from human infections exhibit great genetic diversity, leading to the hypothesis that the genetic background of the infecting strain at least partially determines a patient’s clinical course. However, although certain strains of C. difficile have been suggested to be associated with increased severity, strain typing alone has proved insufficient to explain infection severity. The limited explanatory power of strain typing has been hypothesized to be due to genetic variation within strain types, as well as genetic elements shared between strain types. Homologous recombination is an evolutionary mechanism that can result in large genetic differences between two otherwise clonal isolates, and also lead to convergent genotypes in distantly related strains. More than 400 C. difficile genomes were analyzed here to assess the effect of homologous recombination within and between C. difficile clades. Almost three-quarters of single nucleotide variants in the C. difficile phylogeny are predicted to be due to homologous recombination events. Furthermore, recombination events were enriched in genes previously reported to be important to virulence and host-pathogen interactions, such as flagella, cell wall proteins, and sugar transport and metabolism. Thus, by exploring the landscape of homologous recombination in C. difficile, we identified genetic loci whose elevated rates of recombination mediated diversification, making them strong candidates for being mediators of host-pathogen interaction in diverse strains of C. difficile.

**IMPORTANCE** Infections with C. difficile result in up to half a million illnesses and tens of thousands of deaths annually in the United States. The severity of C. difficile illness is dependent on both host and bacterial factors. Studying the evolutionary history of C. difficile pathogens is important for understanding the variation in pathogenicity of these bacteria. This study examines the extent and targets of homologous recombination, a mechanism by which distant strains of bacteria can share genetic material, in hundreds of C. difficile strains and identifies hot spots of realized recombination events. The results of this analysis reveal the importance of homologous recombination in the diversification of genetic loci in C. difficile that are significant in its pathogenicity and host interactions, such as flagellar construction, cell wall proteins, and sugar transport and metabolism.

## Abstract (precis)

Infections with C. difficile result in up to half a million illnesses and tens of thousands of deaths annually in the United States. The severity of C. difficile illness is dependent on both host and bacterial factors.

## INTRODUCTION

Clostridium difficile is a significant cause of nosocomial infections and can result in clinical presentations ranging from mild diarrhea to severe colitis, sepsis, and death. C. difficile causes disease when it can colonize and grow in the gastrointestinal tract, most often after antibiotic treatment depletes a patient’s normal gut microbiota (1, 2). Two major toxins, toxin A and toxin B, are associated with the virulence of C. difficile, and their secretion results in the destruction of intestinal epithelial cells and recruitment of inflammatory mediators and neutrophils (2–5). However, other factors, such as adhesins, cell surface capsule proteins, hydrolytic enzymes, and flagella, have also been suggested to play an important role in C. difficile colonization, pathogenesis, and virulence (2, 6).

Five major phylogenetic clades of C. difficile have been described (7), and upward of 100 PCR-ribotypes circulate and cause disease in U.S. hospitals (8). Some studies have suggested that epidemic ribotypes 027 (clade 2) and 078 (clade 5) cause more severe disease than other C. difficile strains (9, 10), while others fail to find an association between ribotype and disease severity (11). Thus, more research is needed to explore pathogen factors important in disease manifestation and heterogeneity in severity.

Bacteria can acquire genetic diversity through vertically inherited mutations, recombination via transformation of free DNA, transduction by phage, and conjugation of plasmids (12). Recombination can either be homologous, in which a genomic region is exchanged for an allelic variant, or nonhomologous, in which accessory genes can be transferred from one organism to another (12). Studying bacterial recombination is important in genomics studies since it can both weaken phylogenetic signals and reveal important selective pressures (13). Genomic hot spots for recombination have been hypothesized to be due to both mechanistic and evolutionary reasons. For example, lower rates of recombination have been observed at the terminus compared to the origin of replication, and conserved core genes often flank highly recombinant genes in Escherichia coli (14). However, recombination events that are disadvantageous to the organism will be lost via negative selection, and the events that remain to be observed are likely to be advantageous or neutral in effect (13).

Homologous recombination has been shown to result in high rates of genetic diversity in adhesion, invasion, and colonization factors, as well as cell membrane/surface structures in various pathogenic bacteria (13, 15–17). These factors each play a role in the pathogenesis and/or host interactions of these pathogens. Elevated levels of genetic diversity in these features could lead to adaptions in virulence, transmission, or evasion of the host immune system. In C. difficile, the S-layer cassette that encodes surface proteins important for its antigenicity has been shown to undergo frequent recombination (13, 18). This study utilizes more than 400 C. difficile whole-genome sequences to unveil the contribution of homologous recombination within and between clades of this pathogen, and to elucidate specific functions associated with higher rates of realized recombination events.

## RESULTS AND DISCUSSION

### Strain selection.

**FIG 1:**
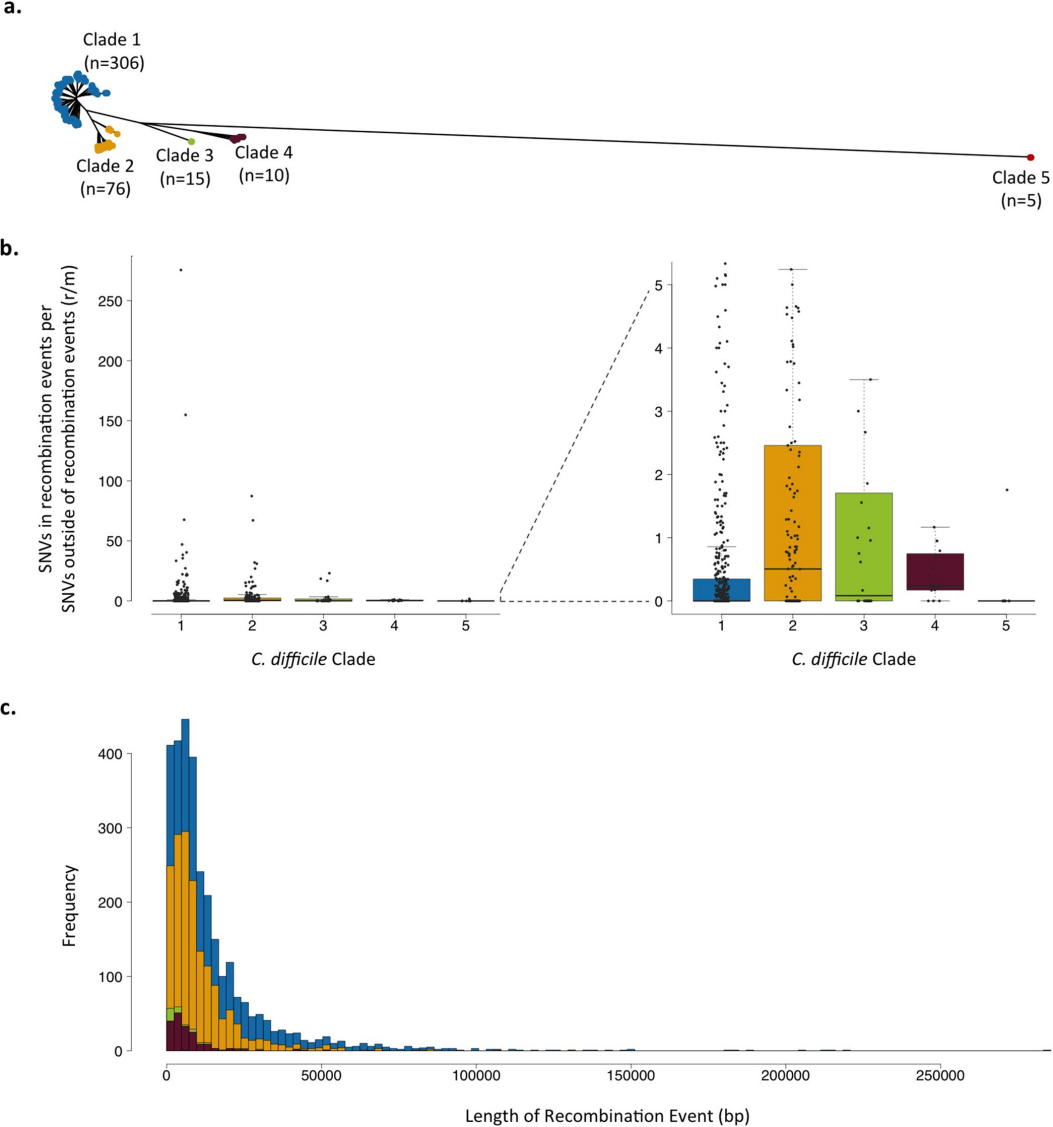
Recombination in five C. difficile clades. (a) Unrooted phylogeny of genomes used in study, colored by previously defined clade designations: clade 1 (blue, *n* = 306), clade 2 (yellow, *n* = 76), clade 3 (green; *n* = 15), clade 4 (purple, *n* = 10), and clade 5 (red, *n* = 5). (b) Distribution of SNVs in recombination events per SNV outside recombination events (r/m) on each branch of each C. difficile clade. Boxplots represent the first to third quartiles of values, with a line representing the median r/m value for each clade, and whiskers are 1.5 times the interquartile range above the third quartile. Values for each branch are plotted on top of the boxplots for each clade. There is a statistically significant difference in r/m values between at least 2 of the clades (Kruskal-Wallis χ^2^_df=4_ = 36.8; *P* < 0.001). (c) Distribution of lengths of recombination events in each C. difficile clade, colored as in panel a.

A total of 412 strains were selected that cluster into five distinct clades (Fig. 1a), as described previously (7). Of the 412 strains, 306 isolates are in clade 1, 76 in clade 2, 15 in clade 3, 10 in clade 2, and 5 in clade 5. Clade 5 is the most distantly related from the rest of the phylogeny, with only 97% sequence identity to reference strain 630 (calculated as number of identical nucleotides over the length of the aligned sequence). Since the majority of isolates are in clades 1 and 2 and since excluding clades 3, 4, and 5 increased the size of the core genome (from 1.66 to 1.75 Mbp) and allowed more genes to be studied, gene and pathway analyses were performed on just the 382 isolates in clades 1 and 2. Moreover, besides ribotype 078, which is in clade 5 and is relatively distantly related to clades 1 and 2, the majority of C. difficile isolates that cause human disease in the United States are in clade 1 (includes ribotype 014/020) and clade 2 (includes ribotype 027) (8), so the results of these analyses can be applicable to much of pathogenic C. difficile.

### Homologous recombination in five *C. difficile* clades.

There is a great deal of heterogeneity in the contribution of recombination to the evolution across the C. difficile phylogeny, as measured by the proportion of single-nucleotide variants (SNVs) inside compared to outside predicted recombination events (r/m) (Fig. 1b). Most branches have more single nucleotide substitution events than recombination events (see Fig. S1 in the supplemental material); however, each recombination event can lead to the transfer of multiple SNVs. Throughout the phylogeny, 72% of SNVs fall within predicted recombination events, even though there are about 15 times as many inferred nucleotide substitution events as there are recombination events. In addition to variance on branches of the tree, there is also as a significant amount of heterogeneity in r/m values between the clades (Kruskal-Wallis χ^2^_df=4_ *P* < 0.001; Fig. 1b), suggesting a potential difference in evolutionary mechanisms between the clades. Clades 2 and 4 have the highest median r/m values, while clades 1 and 5 have the lowest. It is important to note the variance in sample sizes between the clades, which may be biasing these interclade comparisons.

10.1128/mSphere.00799-20.3 FIG S1 Distribution recombination events per single nucleotide substitution event (ρ/θ) on each branch of each C. difficile clade. Boxplots represent the first to the third quartile of values, with a line representing the median ρ/θ value for each clade, and whiskers are 1.5 times the interquartile range above the third quartile. Values for each branch are plotted on top of the boxplots for each clade. There is a statistically significant difference in ρ/θ values between at least of 2 the clades (Kruskal-Wallis χ^2^_df=4_ = 36.8; *P* < 0.001). Colors: clade 1 (blue, *n* = 306); clade 2 (yellow, *n* = 76); clade 3 (green; *n* = 15); clade 4 (purple, *n* = 10); clade 5 (red, *n* = 5). Download FIG S1, PDF file, 0.3 MB. Copyright © 2020 Steinberg and Snitkin. 2020 Steinberg and Snitkin. This content is distributed under the terms of the Creative Commons Attribution 4.0 International license.

The median length of recombination events in all C. difficile clades is ∼6.7 kb, and the maximum length of a predicted recombination event was >100 kb in clade 1. Overall, most recombination events are <10 kb, with an exponential decay in events with increasing length (Fig. 1c). This distribution is observed in all C. difficile clades, though there are more recombination events observed overall in clades 1 and 2 than in clades 3, 4, and 5, likely because the genome sample size is greatest in clades 1 and 2.

### Areas of enriched homologous recombination.

**FIG 2:**
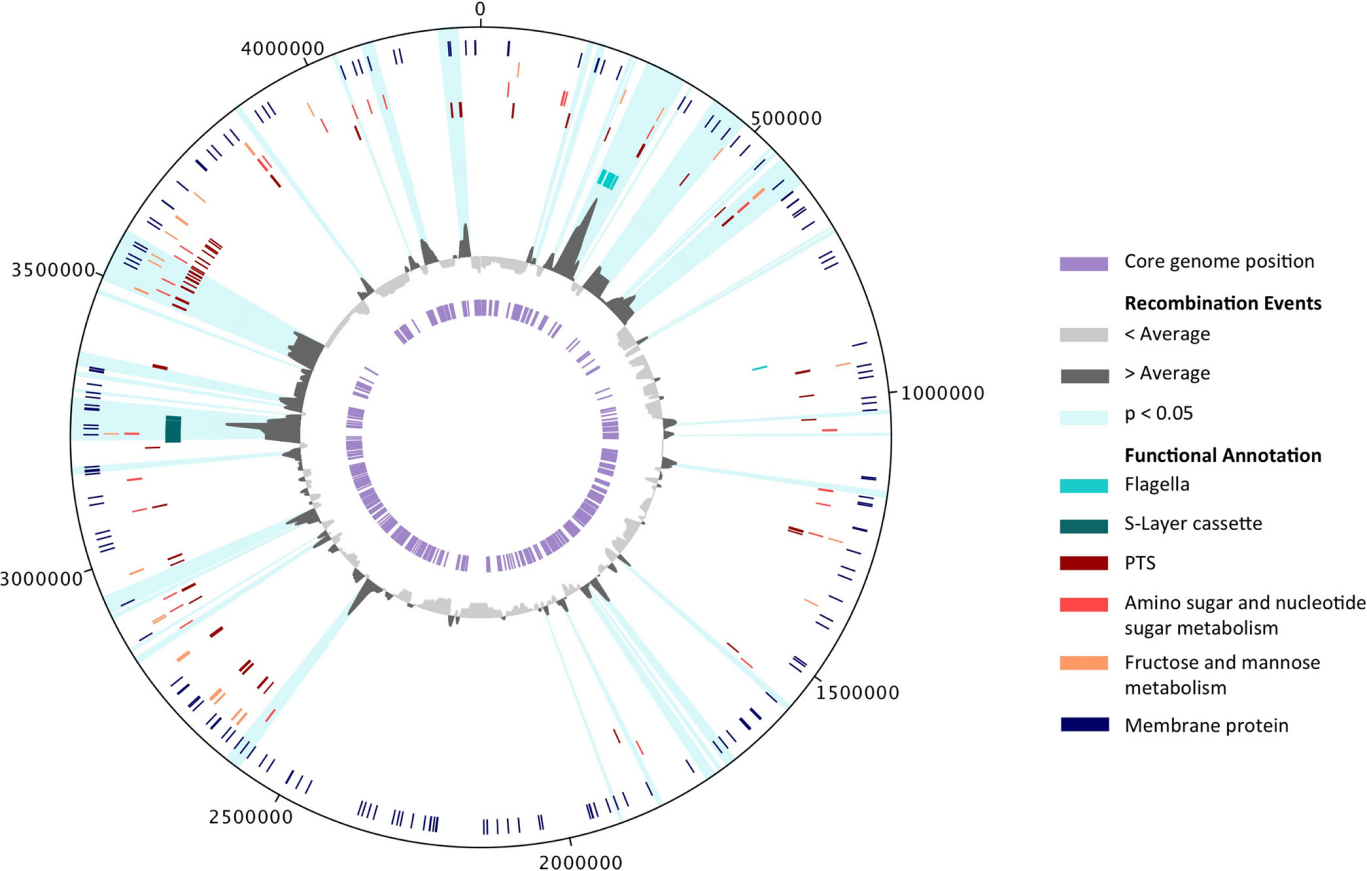
Distribution of recombination events throughout the C. difficile genome. From innermost circle outward: positions in the core genome of clades 1 and 2 (purple) used as input for recombination detection; histogram of recombination events overlapping with each position in the C. difficile genome (dark gray represents more recombination than average, light gray represents less recombination than average); flagellar genes (turquoise); S-layer cassette genes (teal), phosphotransferase system (PTS) genes (dark red); genes involved in amino sugar and nucleotide sugar metabolism (salmon); genes involved in fructose and mannose metabolism (orange), and genes annotated as membrane proteins (navy). Areas highlighted in light blue represent genes that have more recombination than expected with a *P* value of <0.05 as determined by a permutation test. Numbers around the circular genome plot mark the nucleotide position in the reference genome C. difficile 630.

Twelve percent (486/3,980) of C. difficile genes analyzed were significantly enriched for homologous recombination (*P* < 0.05) according to a permutation test (see Table S2 in the supplemental material), and 40 of these genes were enriched for recombination with a fold change of ≥3 (Table 1). The majority of these highly enriched (≥3-fold) genes are involved in flagellar activity. Overall, the set of 486 significant genes were significantly overrepresented in four KEGG pathways, and one COG term involved in flagella, sugar transport and metabolism, and the cell envelope and outer membrane (Table 2). The S-layer cassette, which has previously been shown to be highly recombinant in C. difficile (13, 18), also appears in a high-recombination zone (Fig. 2), with many of the genes involved in S-layer structure significantly enriched for recombination (see Table S2 and Fig. S2 in the supplemental material). There is no KEGG pathway specific to S-layer proteins, but the COG ontology “cell envelope biogenesis, outer membrane,” whose genes are enriched for homologous recombination (Table 2), does include some S-layer proteins.

10.1128/mSphere.00799-20.4 FIG S2 Recombination in the S-layer genes in C. difficile clades 1 and 2. All variants in the S-layer genes of the C. difficile clades 1 and 2 core genome are plotted on the *x* axis based on genomic position. For each genome on the *y* axis, the variant is shown as black (variant present), or white (variant absent). The phylogeny in panel a is a maximum-likelihood tree based on all vertically inherited core variants, and the phylogeny in panel b is a neighbor-joining tree based on only the variants in the S-layer genes. For both panels a and b, clade 1 genomes are shown in shades of green to blue and clade 2 genomes are shown in shades of yellow to red based on their position in the maximum-likelihood phylogeny in panel a. Download FIG S2, PDF file, 2.2 MB. Copyright © 2020 Steinberg and Snitkin. 2020 Steinberg and Snitkin. This content is distributed under the terms of the Creative Commons Attribution 4.0 International license.

**TABLE 1:** C. difficile genes with the greatest increase in recombination events in the clade 1 and 2 core genome

Gene	Annotation	PR events^*a*^	OR events^*b*^	Fold change^*c*^	*P*^*d*^
*fliG*^*e*^	Flagellar motor switch protein FliG	11	58	5.4	<0.001
*fliH*^*e*^	Flagellar assembly protein FliH	11	58	5.3	<0.001
*fliJ*^*e*^	Flagellar protein FliJ	10	52	5.0	<0.001
*fliI*^*e*^	ATP synthase subunit beta FliI	11	52	4.9	<0.001
*fliF*^*e*^	Flagellar MS-ring protein	12	56	4.8	<0.001
*fliE*^*e*^	Flagellar hook-basal body protein FliE	11	50	4.7	<0.001
*flgD*^*e*^	Basal-body rod modification protein FlgD	10	49	4.7	<0.001
*fliK*^*e*^	Flagellar hook-length control protein FliK	10	48	4.6	<0.001
*flgE*^*e*^	Flagellar hook protein FlgE	11	51	4.6	<0.001
*flgB*^*e*^	Flagellar basal-body rod protein FlgB	11	48	4.5	<0.001
*flgC*^*e*^	Flagellar basal-body rod protein FlgC	11	47	4.3	<0.001
CD630_27960^*f*^	Cell surface protein	12	52	4.2	<0.001
*flbD*^*e*^	Flagellar protein FlbD	11	44	4.2	<0.001
*fliS1*^*h*^	Flagellar protein FliS1	11	43	4.0	<0.001
*motB*^*e*^	Flagellar motor rotation protein MotB	11	44	3.9	<0.001
*motA*^*e*^	Flagellar motor rotation protein MotA	11	44	3.9	<0.001
*csrA*^*h*^	Carbon storage regulator CsrA	10	41	3.9	<0.001
*fliL*^*e*^	Flagellar basal body-associated protein FliL	11	41	3.9	<0.001
*fliZ*^*e*^	Flagellar protein FliZ	10	40	3.8	<0.001
*fliS2*^*h*^	Flagellar protein FliS2	11	41	3.8	<0.001
*fliQ*^*e*^	Flagellar biosynthetic protein FliQ	10	40	3.8	<0.001
*flhB*^*e*^	Bifunctional flagellar biosynthesis protein FliR/FlhB	12	45	3.8	<0.001
*fliP*^*e*^	Flagellar biosynthesis protein FliP	11	41	3.7	<0.001
CD630_27970^*f*^	Calcium-binding adhesion protein	17	62	3.7	<0.001
CD630_36440	Hypothetical protein	11	40	3.6	<0.001
*flhA*^*e*^	Flagellar biosynthesis protein FlhA	12	42	3.5	<0.001
*fliC*^*h*^	Flagellin C	11	39	3.4	<0.001
CD630_30830^*i*^	PTS operon transcription antiterminator	11	38	3.4	<0.001
*fliD*^*h*^	flagellar hook-associated protein FliD	12	40	3.3	<0.001
CD630_02380^*h*^	hypothetical protein	11	35	3.3	<0.001
*fliW*^*h*^	Flagellar assembly factor FliW	11	34	3.2	<0.001
*flhF*^*e*^	Flagellar biosynthesis regulator FlhF	11	36	3.1	<0.001
CD630_02410^*g*^	Phosphoserine phosphatase	10	32	3.1	<0.001
CD630_02420^*g*^	Hypothetical protein	10	32	3.1	<0.001
CD630_02430^*g*^	Hypothetical protein	10	32	3.1	<0.001
CD630_02440^*g*^	CDP-glycerol:poly(glycerophosphate) glycerophosphotransferase	10	32	3.1	<0.001
CD630_22420	Hypothetical protein	11	32	3.0	<0.001
*flgL*^*h*^	Flagellar hook-associated protein FlgL	11	34	3.0	<0.001
CD630_22430^*j*^	Membrane protein	11	32	3.0	<0.001
*fliA*^*e*^	Flagellar operon RNA polymerase σ^28^ factor	11	33	3.0	<0.001

a PR events, the predicted number of overlapping recombination events with each gene, calculated as the mean number of recombination events in each of 10,000 permutations randomly placing the identified recombination events.

b OR events, the observed number of overlapping recombination events with each gene, as identified by Gubbins.

c Ratio (fold change) of observed recombination events compared to predicted recombination events.

d Probability (*P*) that the number of observed recombination events is observed by chance based on 10,000 permutations randomly placing the identified recombination events throughout the clades 1 and 2 core genome.

e Gene in F3 regulon.

f Gene in S layer.

g Gene in F2 regulon.

h Gene in F1 regulon.

i PTS gene.

j Membrane protein gene.

**TABLE 2:** Functional categories enriched for recombination in C. difficile clades 1 and 2

Functional category	Fold enrichment	*P*	
		Unadjusted	Benjamini
KEGG pathway			
Flagellar assembly	7.8	<0.001	<0.001
Phosphotransferase system (PTS)	2.7	<0.001	<0.001
Amino sugar and nucleotide sugar metabolism	3.0	<0.001	<0.001
Fructose and mannose metabolism	2.5	<0.001	0.0086
Pentose and glucuronate interconversions	3.1	0.032	0.28
Pentose phosphate pathway	2.4	0.039	0.28
Bacterial chemotaxis	2.6	0.064	0.38
			
COG ontology			
Cell envelope biogenesis, outer membrane	3.1	<0.001	<0.001
Defense mechanisms	1.6	0.078	0.66

10.1128/mSphere.00799-20.1 TABLE S1 Genomes used in analyses and GenBank accession numbers. Download Table S1, CSV file, 0.04 MB. Copyright © 2020 Steinberg and Snitkin. 2020 Steinberg and Snitkin. This content is distributed under the terms of the Creative Commons Attribution 4.0 International license.

10.1128/mSphere.00799-20.2 TABLE S2 All C. difficile genes with statistically significant enrichment of recombination events in the clades 1 and 2 core genome. Download Table S2, XLSX file, 0.1 MB. Copyright © 2020 Steinberg and Snitkin. 2020 Steinberg and Snitkin. This content is distributed under the terms of the Creative Commons Attribution 4.0 International license.

The genomic region from approximately 2.6 to 0.7 Mb appears to be enriched for recombination compared to the other half of the genome (Fig. 2). This could suggest the mechanistic feasibility of recombination in this area and/or grouping of genes where genetic diversity is more evolutionarily advantageous. Low recombination at the terminus of the genome is consistent with findings in Escherichia coli (14).

### Flagella.

**FIG 3:**
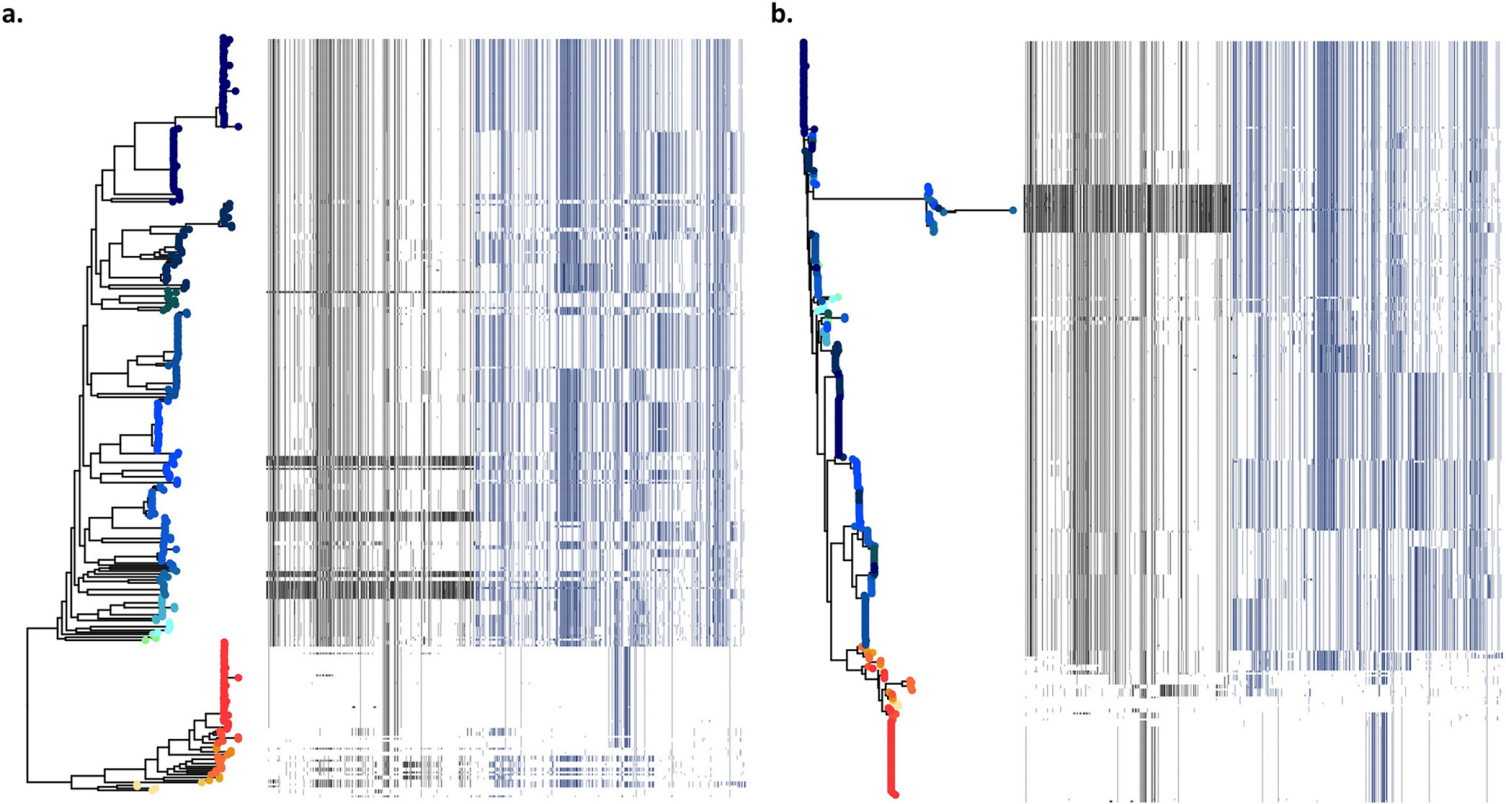
Recombination in flagellar genes in C. difficile clades 1 and 2. All variants in the flagellar region of the C. difficile clades 1 and 2 core genome are plotted on the *x* axis based on genomic position. For each genome on the *y* axis, the variant is shown as black (F1 gene variant present), blue (F3 gene variant present), or white (variant absent). (a and b) The phylogeny in panel a is a maximum-likelihood tree based on all vertically inherited core variants, and the phylogeny in panel b is a neighbor-joining tree based on only the variants in the flagellar region. For panels a and b, clade 1 genomes are shown in shades of green to blue and clade 2 genomes are shown in shades of yellow to red based on their position in the maximum-likelihood phylogeny presented in panel a.

There have been reports of large-scale structural variations of flagella genes in C. difficile, but the contribution of homologous recombination is not as well described. It is clear that unique flagellar variant patterns are present in distantly related isolates (Fig. 3). In particular, within each clade there appears to be a set of distinct flagellar genotypes distributed sporadically throughout the phylogeny, suggesting that large regions of the flagella operons may have been swapped in homologous recombination events. Further, there appears to be a distinct recombination pattern in the two flagellar regulatory units present in the clades 1 and 2 core genome, with there being more F3 genotypes than F1 genotypes present in these isolates. The F3 and F1 operons are responsible for early-stage and late-stage flagellar genes, respectively (19). F1 genes, such as *fliC* and *fliD*, code for the flagellin filament and protein cap (19) that may be involved in protein-protein interactions and recombinant FliC proteins have been shown to be involved in C. difficile immunogenicity (20). F3 genes are associated with other structural components of the flagella, such as the basal body, hook, and motor proteins (19), and the F3 gene *fliA* (*sigD*) has been suggested to be important not only in regulating late-stage flagellar genes but also in the production of C. difficile toxins (21).

Genomic structural diversity of C. difficile flagella has been described before. For instance, C. difficile ribotype 078 (clade 5) strains lack the F3 regulon completely, rendering that ribotype immotile (19). Furthermore, many strains of C. difficile also have an F2 operon between F1 and F3, although these operational units are so diverse that they do not show up in the core genome for clades 1 and 2, and thus homologous recombination in the F2 operon was not analyzed in this study. Supporting the existence of selective pressure for diversification of F2 are previous reports of nonhomologous recombination in this region. C. difficile 630, for example, has four genes in its F2 region, including genes involved in glycosylation, while ribotype 027 isolate CD196 has six seemingly different genes in this area (19, 22).

These results suggest that homologous recombination, in addition to nonhomologous recombination, may be an important mechanism of flagellar diversity in C. difficile. It has been reported that flagella may have a role in motility, colonization, adherence, virulence, and immunogenicity in mucosal pathogens, including C. difficile (23), and *in vivo* studies show that mutants of late-stage flagellar genes *fliC* and *fliD* in C. difficile 630 exhibited greater virulence than did the wild type (24). The enrichment in flagellar diversity in closely related strains observed here suggests an evolutionary advantage, such as antigenic diversification or a unique role in virulence or pathogenicity. Of note, five genes involved in type IV pilus biosynthesis were also shown to be significantly enriched for homologous recombination (see Table S2), which suggests a further role of homologous recombination in the diversification of cell surface structures that act as colonization factors in C. difficile (25).

### PTS and sugar metabolism.

The phosphotransferase system (PTS) is responsible for the active import of certain sugars into bacteria (26). It involves many transmembrane proteins that interact with the extracellular environment, and could be important in host interactions and immunogenicity. The combination of enriched recombination in both PTS and the metabolism of certain sugars (fructose, mannose, and pentose; Table 2) also suggests that recombination may play a role in the ability of C. difficile strains to occupy unique niches via diverse metabolic capabilities. Interestingly, a pleiotropic effect of *fliC* on genes involved in a number of mechanisms, including PTS and carbon metabolism has been observed (6). Variation in the interaction of flagella, PTS, and metabolism proteins itself may be being selected for via homologous recombination.

### Cell membrane and the S layer.

Genes that code for components on the outer layers of bacteria that interact with the outside world and host environment have previously been shown to undergo a relatively large amount of homologous recombination (13). That appears to be the case here as well, since flagella are environment-exposed appendages, and the PTS system involves a number of transmembrane proteins that regulate what sugars can come into the cell. The COG ontology that includes genes involved in cell envelope biogenesis and the outer membrane, including S-layer proteins, is significantly enriched for recombination (Table 2). These data are in line with previous studies that show high recombination in the S-layer surface proteins of C. difficile (18). The majority of genes involved in the S layer (27) were significantly enriched for homologous recombination in this study (Table 1; see also Table S2 and Fig. S2 in the supplemental material).

### Conclusions.

Homologous recombination is an important contributor to the evolution of C. difficile, accounting for an estimated 72% of SNVs. Furthermore, there are genomic regions and functional pathways especially enriched for homologous recombination. Genes involved in the production of proteins that interact with the host and outside environment (i.e., flagella, cell surface proteins, and PTS transporters) and genes important in the metabolism and import of certain sugars are more likely to be highly recombinant. The genes that have been shown here to undergo high rates of homologous recombination have also been documented elsewhere to be associated with toxin production, virulence, and pathogenicity (2, 6, 19–21, 23, 28). These data suggest that homologous recombination may play a significant role in intraclade variation in virulence. There are a number of hypothetical proteins seen here with recombination much higher than expected (Table 1; see also Table S2). These genes should be further investigated in order to determine their potential role in C. difficile pathogenesis and/or virulence, since there appears to be an evolutionary advantage to sustained heterogeneity in these proteins throughout clinically important strains of the bacteria.

Previous work that explored the impact of homologous recombination on C. difficile highlighted the enrichment of recombination in the S layer (13) but failed to find significance in other areas, such as the flagella and PTS. Limitation to only clades 1 and 2 in our analyses allowed for an increased core genome that includes many important genes in these systems that would be missed if only working with the entire C. difficile core genome.

Another implication of this research is the importance of examining the contribution of homologous recombination and allele variants when designing vaccine and drug targets. Both flagellar proteins and S-layer proteins have been suggested as potential vaccine targets for C. difficile (29); however, since the genomic evidence reveals multiple allelic types of these structures, multivalent vaccines may need to be considered.

The analysis of homologous recombination can be conducted on publicly available genomes, and reveal a great deal about pathogen evolution and selective pressures. Here, it has suggested a potentially important role of genetic diversity in flagella, the S layer, PTS, and metabolic pathways to the survival and pathogenesis of C. difficile. Further *in vitro* and *in vivo* assays should be conducted to confirm these roles.

## MATERIALS AND METHODS

### Strain selection.

C. difficile isolates with whole-genome sequence data were selected from a set of 917 genomes previously analyzed in our lab and 4,443 previously published genomes (30) to maximize representation of clinically important strains. To reduce redundancy of nearly identical genomes, isolates that were differentiated from another isolate in the sample by less than six coding mutations were excluded. All genomes used in this analysis were sequenced previously and are publicly available (see Table S1).

### Genome alignments.

Whole-genome alignments were created for each clade, as well as the combination of clades 1 and 2 and the entire set of genomes. Whole-genome alignments were produced as previously described (31). Briefly, each assembly was first concatenated into a pseudochromosome by using mauve contig mover to order and orient large contigs relative to a reference (32). The finished chromosome of the C. difficile 630 strain (included in clade 1) was used as a reference genome where possible, and the most complete whole-genome sequence in each alignment (determined by minimum number of contigs) was chosen as the reference genome otherwise. Each pseudochromosome was aligned to the reference genome using nucmer, and variants were identified relative to the reference genome using the show-snps command (33). Single-nucleotide variants (SNVs) were filtered to remove those SNVs that were likely to be due to alignment or sequencing errors. SNVs were filtered out if (i) they resided in genes annotated as phage, transposase, or integrase; (ii) they resided within 20 bp of the start or end of a contig; (iii) they resided in tandem repeats of total length >20 bp, as determined by the exact-tandem program associated with MUMmer; or (iv) they resided in large inexact repeats as determined by nucmer. Core genomes were calculated based on positions in the reference genome that were aligned in every genome using nucmer.

### Recombination identification.

Gubbins v1.3.4 (Genealogies Unbiased By recom-Binations In Nucleotide Sequences [34]) was used to identify areas likely introduced under homologous recombination in C. difficile. Gubbins was run on core genome alignments of each C. difficile clade, as well as clades 1 and 2 collectively, and on all 412 representative C. difficile genomes used in this study. Genomic regions identified by Gubbins as having densities of SNVs statistically different from background SNV densities in the core genome were inferred to be homologous recombination events, although it is possible that other molecular mechanisms could also have played a role in some of these regions. Maximum-likelihood recombination-filtered phylogenetic trees for the entire set of genomes, as well as just clades 1 and 2, were produced using RAxML v8.2.9 (35) assuming a general time reversible model of nucleotide variations (-m GTRCAT). The bootstrap convergence test and the autoMRE convergence criteria (-N autoMRE) were used to determine the number of bootstrap replicates. The best-scoring maximum-likelihood tree was identified with rapid bootstrap analysis (-f a).

### Identification of genes and pathways with enriched recombination.

A permutation test was implemented and run in R v3.4.0 (36) to randomly place each observed recombination event in the C. difficile clades 1 and 2 core genome. Only clades 1 and 2 were used in this analysis since that is where the majority of recombination events were found and, since these two clades are closely related, it allowed for an expanded core genome compared to the entire set. Furthermore, epidemic ribotypes 014 and 027 fall within clades 1 and 2, respectively. The permutation test was run 10,000 times. Information about all deposited C. difficile genes in the reference genome C. difficile 630 were downloaded from the National Center for Biotechnology Information (NCBI) gene database. A recombination event was considered overlapping with a gene if they overlapped in at least one nucleotide position. One-sided *P* values were calculated for each gene as the number of permutations that predicted a number greater than or equal to the number of recombination events observed plus 1, divided by 10,001. Genes with a *P* value of <0.05 were input as a gene list to the DAVID v6.8 (david.ncifcrf.gov) functional annotation tool to identify the KEGG pathways and COG ontologies associated with increased homologous recombination in C. difficile.

### Data visualization.

An unrooted phylogenetic tree (Fig. 1a) of all 412 isolates was plotted in R v3.4.0 with the ape package v4.1. Box plots and histograms (Fig. 1b and c; see also Fig. S1 in the supplemental material) were made in R v3.4.0. Genome visualization and a recombination map (Fig. 2) was made in DNAPlotter v1.0 (37). Variant heatmaps (Fig. 3; see also Fig. S2) were made with the ggtree package v1.8.1 (38) in R v3.4.0.

### Data availability.

All of the genomes discussed here, as well as their accession numbers, are listed in Table S1 in the supplemental material.

## References

[1] Lessa FC, Mu Y, Bamberg WM, Beldavs ZG, Dumyati GK, Dunn JR, Farley MM, Holzbauer SM, Meek JI, Phipps EC, Wilson LE, Winston LG, Cohen JA, Limbago BM, Fridkin SK, Gerding DN, McDonald LC 2015 Burden of *Clostridium difficile* infection in the United States. N Engl J Med 372:825–834. doi:10.1056/NEJMoa1408913. 25714160 PMC10966662

[2] Borriello SP 1998 Pathogenesis of *Clostridium difficile* infection. J Antimicrob Chemother 41:13–19. doi:10.1093/jac/41.suppl_3.13. 9630370

[3] Borriello SP, Ketley JM, Mitchell TJ, Barclay FE, Welch AR, Price AB, Stephen J 1987 *Clostridium difficile*: a spectrum of virulence and analysis of putative virulence determinants in the hamster model of antibiotic-associated colitis. J Med Microbiol 24:53–64. doi:10.1099/00222615-24-1-53. 3612744

[4] Kuehne SA, Cartman ST, Heap JT, Kelly ML, Cockayne A, Minton NP 2010 The role of toxin A and toxin B in *Clostridium difficile* infection. Nature 467:711–713. doi:10.1038/nature09397. 20844489

[5] Kelly CP, Becker S, Linevsky JK, Joshi MA, O’Keane JC, Dickey BF, LaMont JT, Pothoulakis C 1994 Neutrophil recruitment in *Clostridium difficile* toxin A enteritis in the rabbit. J Clin Invest 93:1257–1265. doi:10.1172/JCI117080. 7907603 PMC294078

[6] Barketi-Klai A, Monot M, Hoys S, Lambert-Bordes S, Kuehne SA, Minton N, Collignon A, Dupuy B, Kansau I 2014 The flagellin FliC of *Clostridium difficile* is responsible for pleiotropic gene regulation during *in vivo* infection. PLoS One 9:e96876. doi:10.1371/journal.pone.0096876. 24841151 PMC4026244

[7] Elliott B, Androga GO, Knight DR, Riley TV 2017 *Clostridium difficile* infection: evolution, phylogeny, and molecular epidemiology. Infect Genet Evol 49:1–11. doi:10.1016/j.meegid.2016.12.018. 28012982

[8] Waslawski S, Lo ES, Ewing SA, Young VB, Aronoff DM, Sharp SE, Novak-Weekley SM, Crist AE, Dunne WM, Hoppe-Bauer J, Johnson M, Brecher SM, Newton DW, Walk ST 2013 *Clostridium difficile* ribotype diversity at six health care institutions in the United States. J Clin Microbiol 51:1938–1941. doi:10.1128/JCM.00056-13. 23554188 PMC3716112

[9] Rao K, Micic D, Natarajan M, Winters S, Kiel MJ, Walk ST, Santhosh K, Mogle JA, Galecki AT, Lebar W, Higgins PDR, Young VB, Aronoff DM 2015 *Clostridium difficile* ribotype 027: relationship to age, detectability of toxins A or B in stool with rapid testing, severe infection, and mortality. Clin Infect Dis 61:233–241. doi:10.1093/cid/civ254. 25828993 PMC4565993

[10] Goorhuis A, Bakker D, Corver J, Debast SB, Harmanus C, Notermans DW, Bergwerff AA, Dekker FW, Kuijper EJ 2008 Emergence of *Clostridium difficile* infection due to a new hypervirulent strain, polymerase chain reaction ribotype 078. Clin Infect Dis 47:1162–1170. doi:10.1086/592257. 18808358

[11] Walk ST, Micic D, Jain R, Lo ES, Trivedi I, Liu EW, Almassalha LM, Ewing SA, Ring C, Galecki AT, Rogers MAM, Washer L, Newton DW, Malani PN, Young VB, Aronoff DM 2012 *Clostridium difficile* ribotype does not predict severe infection. Clin Infect Dis 55:1661–1668. doi:10.1093/cid/cis786. 22972866 PMC3501335

[12] Vos M 2009 Why do bacteria engage in homologous recombination? Evol Microbiol 17:226–232. doi:10.1016/j.tim.2009.03.001. 19464181

[13] Yahara K, Didelot X, Jolley KA, Kobayashi I, Maiden MCJ, Sheppard SK, Falush D 2016 The landscape of realized homologous recombination in pathogenic bacteria. Mol Biol Evol 33:456–471. doi:10.1093/molbev/msv237. 26516092 PMC4866539

[14] Touchon M, Hoede C, Tenaillon O, Barbe V, Baeriswyl S, Bidet P, Bingen E, Bonacorsi S, Bouchier C, Bouvet O, Calteau A, Chiapello H, Clermont O, Cruveiller S, Danchin A, Diard M, Dossat C, Karoui ME, Frapy E, Garry L, Ghigo JM, Gilles AM, Johnson J, Le Bouguénec C, Lescat M, Mangenot S, Martinez-Jéhanne V, Matic I, Nassif X, Oztas S, Petit MA, Pichon C, Rouy Z, Ruf CS, Schneider D, Tourret J, Vacherie B, Vallenet D, Médigue C, Rocha EPC, Denamur E 2009 Organized genome dynamics in the *Escherichia coli* species results in highly diverse adaptive paths. PLoS Genet 5:e1000344. doi:10.1371/journal.pgen.1000344. 19165319 PMC2617782

[15] Didelot X, Guillaume M, Falush D, Darling AE 2012 Impact of homologous and non-homologous recombination in the genomic evolution of *Escherichia coli*. BMC Genomics 13:256. doi:10.1186/1471-2164-13-256. 22712577 PMC3505186

[16] Joseph SJ, Didelot X, Rothschild J, De Vries HJC, Morre SA, Read TD, Dean D 2012 Population genomics of *Chlamydia trachomatis*: insights on drift, selection, recombination, and population structure. Mol Biol Evol 29:3933–3946. doi:10.1093/molbev/mss198. 22891032 PMC3494276

[17] Lüneberg E, Glenn-Calvo E, Hartmann M, Bär W, Frosch M 1998 The central, surface-exposed region of the flagellar hook protein FlgE of *Campylobacter jejuni* shows hypervariability among strains. J Bacteriol 180:3711–3714. doi:10.1128/JB.180.14.3711-3714.1998. 9658019 PMC107344

[18] Dingle KE, Didelot X, Ansari MA, Eyre DW, Vaughan A, Grif D, Ip CLC, Batty EM, Golubchik T, Bowden R, Jolley KA, Hood DW, Fawley WN, Walker AS, Peto TE, Wilcox MH, Crook DW 2013 Recombinational switching of the *Clostridium difficile* S-layer and a novel glycosylation gene cluster revealed by large-scale whole-genome sequencing. J Infect Dis 207:675–686. doi:10.1093/infdis/jis734. 23204167 PMC3549603

[19] Stevenson E, Minton NP, Kuehne SA, Cd CD 2015 The role of flagella in *Clostridium difficile* pathogenicity. Trends Microbiol 23:275–282. doi:10.1016/j.tim.2015.01.004. 25659185

[20] Ghose C, Eugenis I, Sun X, Edwards AN, Mcbride SM, Pride DT, Kelly CP, Ho DD 2016 Immunogenicity and protective efficacy of recombinant *Clostridium difficile* flagellar protein FliC. Emerg Microbes Infect 5:1–10. doi:10.1038/emi.2016.8. PMC4777929 26839147

[21] El Meouche I, Peltier J, Monot M, Soutourina O, Pestel-Caron M, Dupuy B, Pons J-L 2013 Characterization of the SigD regulon of *Clostridium difficile* and its positive control of toxin production through the regulation of *tcdR*. PLoS One 8:e83748. doi:10.1371/journal.pone.0083748. 24358307 PMC3865298

[22] Stabler RA, He M, Dawson L, Martin M, Valiente E, Corton C, Lawley TD, Sebaihia M, Quail MA, Rose G, Gerding DN, Gibert M, Popoff MR, Parkhill J, Dougan G, Wren BW 2009 Comparative genome and phenotypic analysis of *Clostridium difficile* 027 strains provides insight into the evolution of a hypervirulent. Genome Biol 10:R102. doi:10.1186/gb-2009-10-9-r102. 19781061 PMC2768977

[23] Ramos HC, Rumbo M, Sirard J 2004 Bacterial flagellins: mediators of pathogenicity and host immune responses in mucosa. Trends Microbiol 12:510–517. 10.1016/j.tim.2004.09.002 15488392

[24] Dingle TC, Mulvey GL, Armstrong GD 2011 Mutagenic analysis of the *Clostridium difficile* flagellar proteins, FliC and FliD, and their contribution to virulence in hamsters. Infect Immun 79:4061–4067. doi:10.1128/IAI.05305-11. 21788384 PMC3187235

[25] McKee RW, Aleksanyan N, Garrett EM, Tamayo R 2018 Type IV pili promote *Clostridium difficile* adherence and persistence in a mouse model of infection. Infect Immun 86:1–13. doi:10.1128/IAI.00943-17. PMC5913833 29483294

[26] Postma PW, Lengeler JW 1985 Phosphoenolpyruvate: carbohydrate phosphotransferase system of bacteria. Microbiol Rev 49:232–269. doi:10.1128/MMBR.49.3.232-269.1985. 3900671 PMC373035

[27] Bradshaw WJ, Roberts AK, Shone CC, Acharya KR 2018 The structure of the S-layer of *Clostridium difficile*. J Cell Commun Signal 12:319–331. doi:10.1007/s12079-017-0429-z. 29170885 PMC5842191

[28] Baban ST, Kuehne SA, Barketi-Klai A, Cartman ST, Kelly ML, Hardie KR, Kansau I, Collignon A, Minton NP 2013 The role of flagella in *Clostridium difficile* pathogenesis: comparison between a non-epidemic and an epidemic strain. PLoS One 8:e73026. doi:10.1371/journal.pone.0073026. 24086268 PMC3781105

[29] Péchiné S, Bruxelle JF, Janoir C, Collignon A 2018 Targeting *Clostridium difficile* surface components to develop immunotherapeutic strategies against *Clostridium difficile* infection. Front Microbiol 9:1009. doi:10.3389/fmicb.2018.01009. 29875742 PMC5974105

[30] Dingle KE, Didelot X, Quan TP, Eyre DW, Stoesser N, Golubchik T, Harding RM, Wilson DJ, Griffiths D, Vaughan A, Finney JM, Wyllie DH, Oakley SJ, Fawley WN, Freeman J, Morris K, Martin J, Howard P, Gorbach S, Goldstein EJC, Citron DM, Hopkins S, Hope R, Johnson AP, Wilcox MH, Peto TEA, Walker AS, Crook DW, Del Ojo Elias C, Crichton C, Kostiou V, Giess A, Davies J, Modernising Medical Microbiology Informatics Group 2017 Effects of control interventions on *Clostridium difficile* infection in England: an observational study. Lancet Infect Dis 17:411–421. doi:10.1016/S1473-3099(16)30514-X. 28130063 PMC5368411

[31] Snitkin ES, Zelazny AM, Gupta J, Comparative N, Program S, Palmore TN, Murray PR, Segre JA, NISC Comparative Sequencing Program 2013 Genomic insights into the fate of colistin resistance and *Acinetobacter baumannii* during patient treatment. Genome Res 23:1155–1162. doi:10.1101/gr.154328.112. 23564252 PMC3698508

[32] Rissman AI, Mau B, Biehl BS, Darling AE, Glasner JD, Perna NT 2009 Reordering contigs of draft genomes using the Mauve Aligner. Bioinformatics 25:2071–2073. doi:10.1093/bioinformatics/btp356. 19515959 PMC2723005

[33] Delcher AL, Phillippy A, Carlton J, Salzberg SL 2002 Fast algorithms for large-scale genome alignment and comparison. Nucleic Acids Res 30:2478–2483. doi:10.1093/nar/30.11.2478. 12034836 PMC117189

[34] Croucher NJ, Page AJ, Connor TR, Delaney AJ, Keane JA, Bentley SD, Parkhill J, Harris SR 2015 Rapid phylogenetic analysis of large samples of recombinant bacterial whole-genome sequences using Gubbins. Nucleic Acids Res 43:e15. doi:10.1093/nar/gku1196. 25414349 PMC4330336

[35] Stamatakis A 2014 RAxML version 8: a tool for phylogenetic analysis and post-analysis of large phylogenies. Bioinformatics 30:1312–1313. doi:10.1093/bioinformatics/btu033. 24451623 PMC3998144

[36] R Core Team. 2019 R: a language and environment for statistical computing. R Foundation for Statistical Computing, Vienna, Austria https://www.r-project.org/.

[37] Carver T, Thomson N, Bleasby A, Berriman M, Parkhill J 2009 DNAPlotter: circular and linear interactive genome visualization. Bioinformatics 25:119–120. doi:10.1093/bioinformatics/btn578. 18990721 PMC2612626

[38] Yu G, Smith DK, Zhu H, Guan Y, Lam TT 2017 GGTREE: an R package for visualization and annotation of phylogenetic trees with their covariates and other associated data. Methods Ecol Evol 8:28–36. doi:10.1111/2041-210X.12628.

