# Supplementary figures and images for: Homologous Recombination in Clostridioides difficile Mediates Diversification of Cell Surface Features and Transport Systems
Source: mSphere. 2020 Nov 18;5(6):e00799-20. doi: 10.1128/mSphere.00799-20 (PMC7677006; doi:10.1128/mSphere.00799-20)

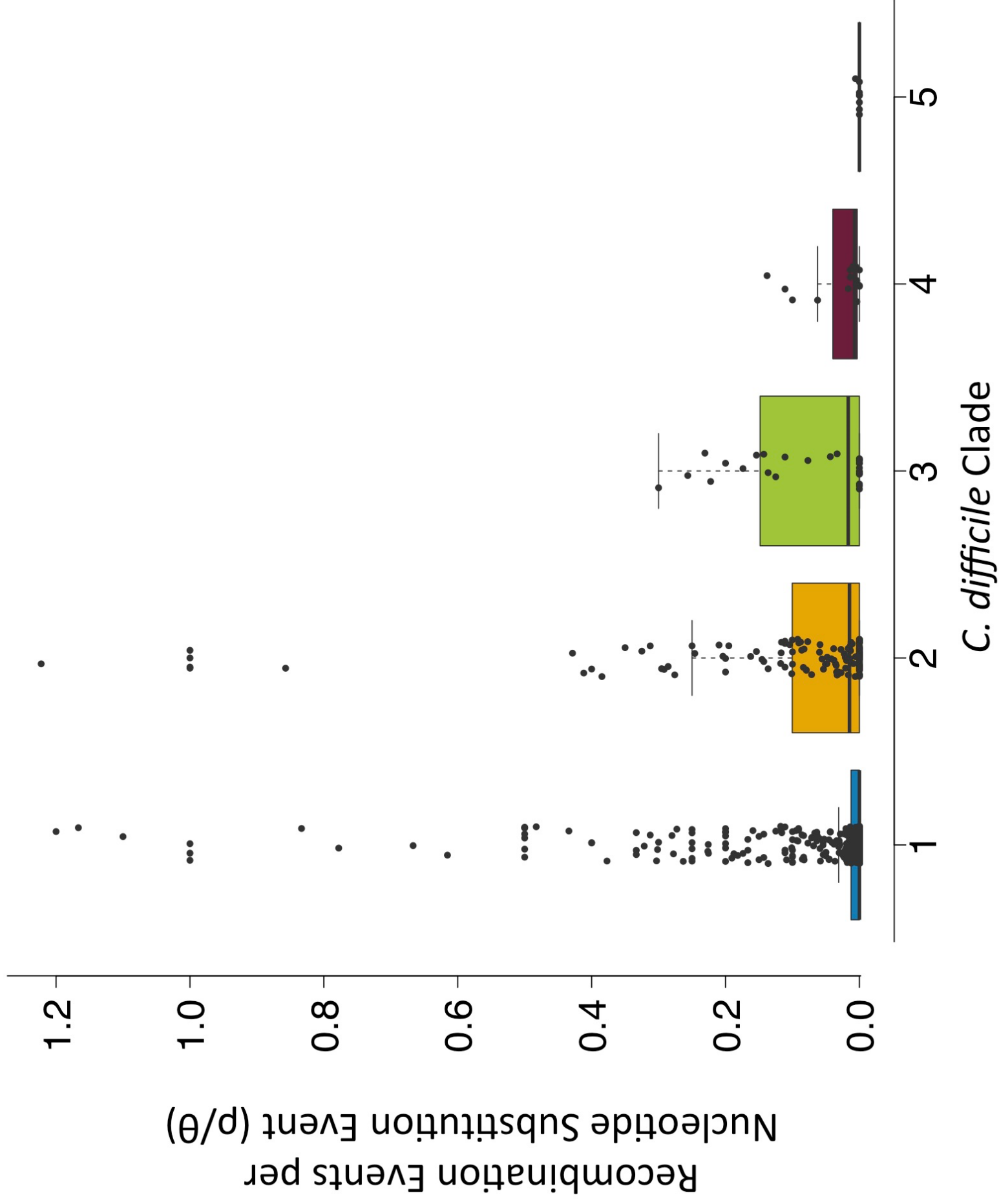

Supplement: FIG S1 [file SM-MSPH200002sf1.pdf]

a.

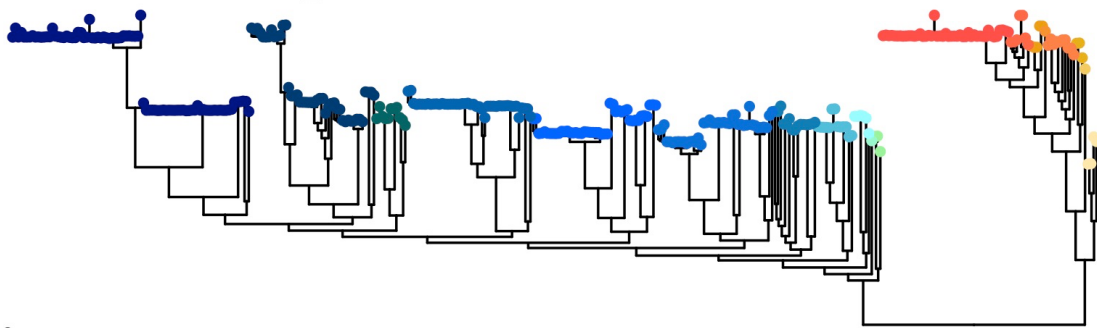

b.

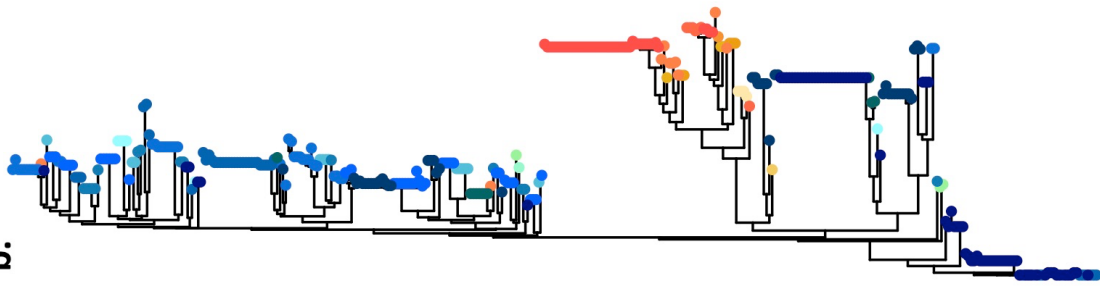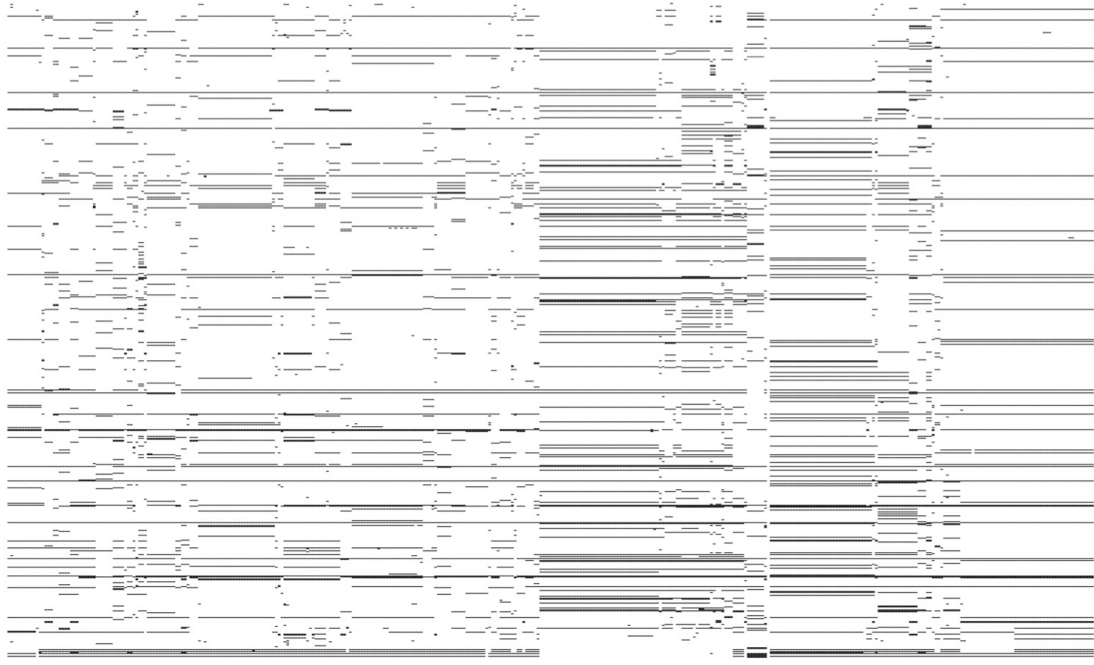

Supplement: FIG S2 [file SM-MSPH200002sf2.pdf]
